# Preconception Physical Exercise Is Associated with Phenotype-Specific Cardiovascular Alterations in Women at Risk for Gestational Hypertensive Disorders

**DOI:** 10.3390/jcm13144164

**Published:** 2024-07-16

**Authors:** Pauline Dreesen, Pauline Volders, Dorien Lanssens, Sandy Nouwen, Birgit Vrancken, Febe Janssen, Bert O. Eijnde, Dominique Hansen, Michael Ceulemans, Adelheid Soubry, Wilfried Gyselaers

**Affiliations:** 1 Faculty of Medicine and Life Sciences, Limburg Clinical Research Center, Hasselt University, 3590 Diepenbeek, Belgiumdorien.lanssens@uhasselt.be (D.L.); wilfried.gyselaers@uhasselt.be (W.G.); 2 Future Health, Ziekenhuis Oost-Limburg, 3600 Genk, Belgium; 3 SMRC Sports Medical Research Center, BIOMED Biomedical Research Institute, Faculty of Medicine & Life Sciences, Hasselt University, 3590 Diepenbeek, Belgium; 4 Division of Sport Science, Faculty of Medicine & Health Sciences, Stellenbosch University, Stellenbosch 7602, South Africa; 5 REVAL Rehabilitation Research Centre, Faculty of Rehabilitation Sciences, Hasselt University, 3590 Diepenbeek, Belgium; dominique.hansen@uhasselt.be; 6 Department of Cardiology, Heart Centre Hasselt, Jessa Hospital, 3500 Hasselt, Belgium; 7 Clinical Pharmacology and Pharmacotherapy, Department of Pharmaceutical and Pharmacological Sciences, KU Leuven, 3000 Leuven, Belgium; michael.ceulemans@kuleuven.be; 8 IQ Health, Radboud University Medical Center, 6525 XZ Nijmegen, The Netherlands; 9 Child & Youth Institute, KU Leuven, 3000 Leuven, Belgium; 10 Epigenetic Epidemiology Lab, Department of Human Genetics, Faculty of Medicine, KU Leuven, 3000 Leuven, Belgium; adelheid.soubry@kuleuven.be; 11 Department of Obstetrics & Gynecology, Ziekenhuis Oost-Limburg, 3600 Genk, Belgium

**Keywords:** physical exercise, maternal hemodynamics, cardiac output, total peripheral resistance, preconception care, gestational hypertensive disorders

## Abstract

**Background/Objectives**: Gestational hypertensive disorders (GHD) pose significant maternal and fetal health risks during pregnancy. Preconception physical exercise has been associated with a lower incidence of GHD, but insights into the cardiovascular mechanisms remain limited. This study aimed to evaluate the effect of preconception physical exercise on the complete cardiovascular functions of women at risk for GHD in a subsequent pregnancy. **Methods**: A non-invasive hemodynamics assessment of arteries, veins, and the heart was performed on 40 non-pregnant women at risk for developing GHD in a subsequent pregnancy. Measurements of an electrocardiogram Doppler ultrasound, impedance cardiography and bio-impedance spectrum analysis were taken before and after they engaged in physical exercise (30–50 min, 3×/week, 4–6 months). **Results**: After a mean physical exercise period of 29.80 weeks, the total peripheral resistance (TPR), diastolic blood pressure and mean arterial pressure decreased in the total study population, without changing cardiac output (CO). However, in 42% (9/21) of women categorized with high or low baseline CO (>P75 or <P25 resp.), a shift in CO was observed towards the normal reference interquartile range (P25–P75). This was associated with improved hepatic venous and central arterial hemodynamic functions. Similar changes in TPR occurred in 38% (11/29) of women classified as having low or high baseline TPR. **Conclusions**: As in pregnancy, output- or resistance-dominant cardiovascular profiles already exist prior to conception. This study illustrates that preconception physical exercise shifts high or low CO and/or TPR towards the normal midrange, allowing women at risk for GHD to start a subsequent pregnancy with a more gestation-adaptable cardiovascular system.

## 1. Introduction

Uncomplicated pregnancies are associated with adaptations of the maternal heart, arteries, and veins that create optimal conditions for the growth and development of the fetus without compromising the mother’s health. These hemodynamic changes involve a uniform vasodilatation, and a subsequent increase in cardiac output (CO) and a decrease in total peripheral resistance (TPR), resulting in high-volume low-resistance circulation [1]. However, in pregnancies complicated by gestational hypertensive disorders (GHD), these maternal cardiovascular (CV) adaptations are deviating, affecting 5–10% of pregnancies worldwide [1,2,3]. GHD encompass a heterogeneous group of syndromes, including chronic or essential hypertension, gestational hypertension (GH), and pre-eclampsia (PE). The latter can be further classified as early-onset PE (EPE) or late-onset PE (LPE) if diagnosed before or after 34 weeks of gestation, respectively [4]. Previous research revealed that different subtypes of GHD show different CV phenotypes based on their underlying CO and TPR level, which can be classified as being volume- or resistance-dominant [5,6].

A wide range of complications are associated with GHD, varying in severity from mild hypertension without any other symptoms to multisystem conditions with potentially life-threatening consequences for both mother and fetus [1,4]. Several risk factors are associated with the development of GHD, such as a high pre-pregnancy body-mass index (BMI > 30 kg/m^2^), advanced maternal age (≥40 years old), a multiple pregnancy, maternal smoking, in vitro fertilization, a family history of hypertension, previous PE, and maternal co-morbidities (i.e., pre-gestational diabetes mellitus, chronic hypertension, chronic kidney disease, and systemic lupus erythematosus) [4,7]. Additionally, the incidence of GHD is rising due to the increasing prevalence of cardiometabolic diseases among younger women [8]. The risk of developing GHD can be managed by preventive measures during pregnancy such as remote blood pressure monitoring and low-dose aspirin use starting at or before 16 weeks of gestation [4,9]. Furthermore, women who adhere to the recommended aerobic training guidelines during pregnancy (60–150 min/week) have up to 30% less risk for developing GHD [10]. Moreover, it has been shown that women who exercise before conception and in early pregnancy have the greatest reduction in risk for GHD [10,11,12,13]. Women performing exercise prior to pregnancy had a 22–35% relative risk reduction in developing PE [10]. However, these studies merely describe the association between physical exercise and the incidence of GHD or the effect of physical exercise on a subset of hemodynamic parameters rather than the overall CV profile [10,13]. As such, important aspects of the preventive potential of physical exercise on specific aspects of the CV system remain unexplored. The primary aim of this study was to evaluate the effect of physical exercise on the complete CV profile of non-pregnant women who are at risk for developing GHD in a subsequent pregnancy, and to verify whether distinct CV phenotypes also exist prior to conception in this at-risk population. New insights can lead to personalized exercise programs based on their underlying CV profile. This proactive approach allows women to improve their CV system prior to conception, enabling them to start subsequent pregnancies with a more favorable CV profile.

## 2. Materials and Methods

This retrospective study was conducted at Ziekenhuis Oost-Limburg (ZOL, Genk, Belgium). Ethical approval was obtained from the institutional review board of the local ethical committee of Ziekenhuis Oost-Limburg (reference: Z-2021055).

### 2.1. Study Population

Non-pregnant women (age ≥ 18 years) who are at risk for developing GHD in a subsequent pregnancy and who underwent a CV assessment before and after receiving the advice to engage in physical exercise as part of usual care between May 2016 and February 2023 were included for analysis. An increased risk for GHD was defined by the presence of one or more of the following risk factors: essential hypertension, prior pregnancy complications, a family history of GHD, a low maternal birth weight, a high BMI (>30 kg/m^2^), an advanced maternal age (≥40 years old) or other relevant co-morbidities such as thrombophilia, kidney diseases, diabetes mellitus, or auto-immune disorders in women trying to conceive [4,7].

### 2.2. Cardiovascular Assessment

**Figure 1:**
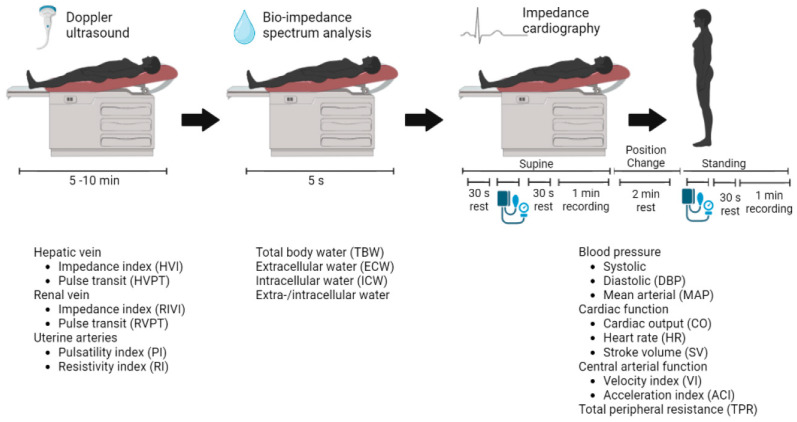
Standardized validated cardiovascular assessment protocol. The non-invasive techniques used are (from left to right): Electrocardiogram-Doppler ultrasound and bio-impedance spectrum analysis in a supine position, followed by impedance cardiography in a supine and a standing position. The resulting parameters are presented with each technique, respectively.

The CV assessments were performed at Ziekenhuis Oost-Limburg as part of routine clinical care. Three non-invasive techniques were combined to gather information about the arteries, heart, veins, and body fluid status according to a reported standardized validated CV assessment protocol (Figure 1) [6].

Electrocardiogram (ECG)-assisted Doppler ultrasonography was used to examine Doppler signals at the hepatic and renal veins and the uterine arteries (Aplio Mx, Toshiba Medical Systems nv, Sint-Stevens-Woluwe, Belgium). Bio-impedance spectrum analysis using a multiple-frequency bioelectrical impedance analyzer (Maltron BioScan 920-II, Maltron International, Essex, UK) provided information about the body fluid status. Impedance cardiography (ICG) using the Non-Invasive Continuous Cardiac Output Monitor (NICCOMO, Medis Medizinische Messtechnik, Ilmenau, Germany) registered the following heart parameters in the supine and the standing positions: (1) cardiac function parameters including stroke volume (SV) in mL, heart rate (HR) in beats per minute, and CO in L/min, were calculated using the formula of Bernstein (CO = HR × SV); (2) central arterial function parameters such as the aorta flow velocity index (VI) in 1/1000/s and the acceleration index (ACI) in 1/100/s^2^; and (3) TPR was calculated as the mean arterial pressure (MAP) multiplied by 80 and divided by CO, expressed in dyn∙s∙cm^−5^. Arterial blood pressure was recorded using automated sphygmomanometry incorporated in the NICCOMO device [6].

The biological nature and interpretation of the collected CV parameters are presented in Table S1. All parameters were recorded in a resting phase at the baseline preconception visit (PC) and at the preconception visit following the advice to perform physical exercise (defined as the preconception post-sport visit (PCPS)). The PC-PCPS interval is considered a measure for the duration of the physical exercise.

### 2.3. Physical Exercise

The non-pregnant women were given the advice to exercise 30–50 min at least three times per week for approximately four to six months. They were encouraged to perform aerobic exercise (such as cycling, running, walking, swimming, etc.), as this regimen could easily be continued during subsequent pregnancy, which is in line with the International Society for the Study of Hypertension in Pregnancy guidelines for exercise during pregnancy [4]. Although the advice consisted of aerobic exercise, any type of physical exercise was accepted since the focus was to abolish a sedentary lifestyle.

### 2.4. Data Collection

Demographic and clinical characteristics such as age, BMI, parity, the indication for the CV measurement, co-morbidities, a family history of cardiovascular disease, and medication usage were collected using a computerized system (Castor EDC).

### 2.5. Statistics

The mean ± standard deviation (SD) or the median with interquartile range (IQR) were calculated for normally distributed and skewed distributions, respectively. Given that CO and TPR are key parameters in predicting pregnancy outcomes [5], the study population was categorized according to baseline CO and TPR percentiles (<P25; P25–P75; >P75) into the following profiles: low CO (<5.0 L/min; <P25), normal CO (5.0–6.5 L/min; P25–P75) or high CO (>6.5 L /min; >P75), and low TPR (<1149 dyn∙s∙cm^−5^; <P25), normal TPR (1149–1537 dyn∙s∙cm^−5^; P25–P75), or high TPR (>1537 dyn∙s∙cm^−5^; >P75). Categorical data were reported as absolute values and proportions. Demographic data were compared among the three CO or TPR groups (low, normal, high) using one-way ANOVA for mean comparisons, the Kruskal–Wallis test for median comparisons, and the Chi-square test or Fisher’s Exact test for proportion comparisons. Pre- and post-exercise parameters were compared using a two-sided paired Student’s *t*-test for means and the non-parametric Wilcoxon signed-rank test for medians. A Spearman’s rho correlation analysis was performed to evaluate the association between the PC-PCPS interval and the change in CO or TPR level. A significance level of 5% (*p* < 0.05) was considered statistically significant. For categorical data, a more stringent significance level of 1.7% (*p* < 0.017) was applied due to multiple testing. Statistical analysis was performed with Statistical Package for Social Sciences release 28.0.1.1 (IBM SPSS Statistics, Chicago, IL, USA).

## 3. Results

### 3.1. Study Population

**Figure 2:**
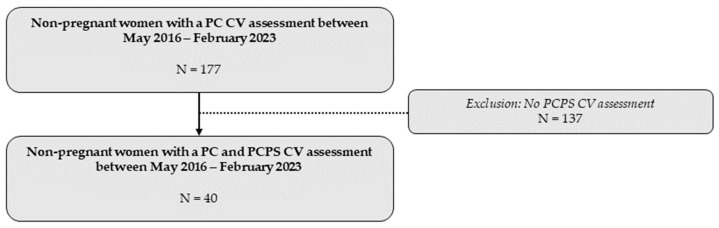
Overview of the total study population included in the statistical analysis. CV: cardiovascular; PC: preconception; PCPS: preconception post-sport.

A total of 40 women underwent a CV assessment before and after receiving the advice to engage in physical exercise at Ziekenhuis Oost-Limburg between May 2016 and February 2023 (Figure 2).

Detailed demographic characteristics of the study population are presented in Table 1. The study population mainly consisted of multiparous women (80.00%). The most common reasons for referral to a CV assessment were previous pregnancy complications (82.50%) and chronic hypertension (30.00%).

### 3.2. Preconception Physical Exercise in the Study Population

The mean (±SD) time between the PC and PCPS visit was 29.80 (±17.86) weeks. A statistically significant decrease in TPR (1543 vs. 1410 dyn∙s∙cm^−5^, *p* = 0.016), diastolic blood pressure (DBP:92 vs. 88 mmHg, *p* = 0.009), and MAP (103 vs. 99 mmHg, *p* = 0.030) was observed after the preconception physical exercise advice. The other collected CV parameters showed no statistically significant difference between both visits (Table S2).

### 3.3. Phenotype-Specific Cardiovascular Changes

If subjects were categorized according to baseline CO values, 52.50% (n = 21) showed an aberrant CO profile (i.e., high or low) before receiving the physical exercise advice (Table S3). This was the case for 75.50% (n = 29) of the study population if categorization was based on baseline TPR values, with a high baseline TPR profile being the most numerous group (52.50%) (Table S4).

Demographic characteristics of the subgroups categorized by baseline CO and TPR values are presented in Table S5 and Table S6, respectively.

#### 3.3.1. Baseline CO Profiles

**Figure 3:**
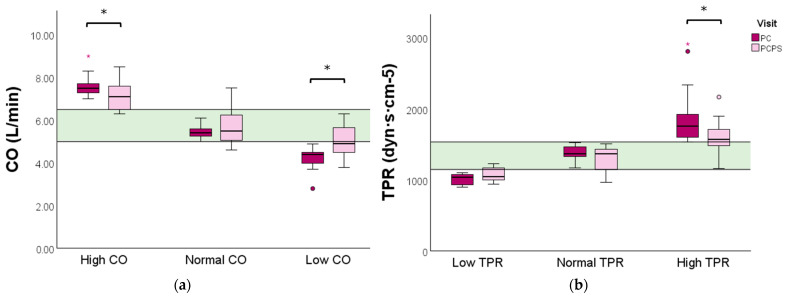
CO and TPR levels evaluated before and after preconception physical exercise advice for each CV profile. The study population (n = 40) was categorized into (**a**) low (n = 12), normal (n = 19), or high (n = 9) baseline CO profiles, and (**b**) high (n = 21), normal (n = 11), or low (n = 8) baseline TPR profiles. Data are presented as boxplots with a median and IQR. The green bar represents the normal reference range (P25–P75) for CO and TPR of non-pregnant women, respectively. CO: cardiac output; TPR: total peripheral resistance; PC: preconception; PCPS: preconception post-sport. * *p* < 0.05. ° Outlier. Red * extreme outlier.

Women with a high baseline CO profile experienced a significant reduction in CO (7.5 vs. 7.1 L/min, *p* = 0.041) following the physical exercise advice. In comparison, a significant increase in CO was observed in the low baseline CO profile group (4.4 vs. 4.9 L/min, *p* = 0.009) (Figure 3a and Table S3).

Further analysis of the post-sport CV system parameters revealed that women with a high baseline CO profile exhibited a significant increase in hepatic vein impedance index (HVI: 0.091 vs. 1.30, *p* = 0.038) and in pulsatility index of the left uterine artery (L Aut PI: 1.39 vs. 1.55, *p* = 0.038). On the other hand, women with a low baseline CO profile experienced a significant increase in aorta flow acceleration and velocity indices (ACI: 121 vs. 163 1/100/s2, *p* = 0.015 and VI: 58 vs. 69 1/1.000/s, *p* = 0.042, respectively). Moreover, a significant increase in the left renal interlobar vein impedance index (L RIVI) was found in women with a normal baseline CO profile (0.43 vs. 0.50, *p* = 0.017) (Table S3).

**Figure 4:**
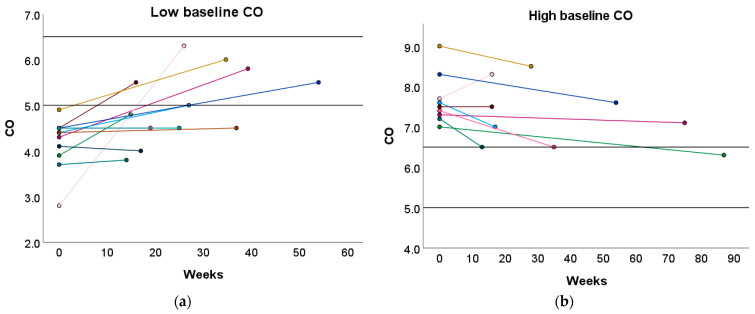
Change in CO following the preconception physical exercise advice at the subject level. The individual change in CO between the preconception visit and the post-sport measurement is presented in function of the weeks between the measurements for women with (**a**) a low baseline CO profile and (**b**) a high baseline CO profile. Each colored dot represents an individual subject. Each colored line refers to the change in CO for an individual subject. The black horizontal lines represent the upper and lower limit of the normal reference interquartile range (P25–P75). CO: cardiac output.

Furthermore, 33.33% and 50.00% of women with a high or low baseline CO profile transitioned towards the normal reference interquartile range (P25–P75) after the preconception physical exercise advice (Figure 4 and Table S7). In contrast, the recommended physical exercise did not induce a significant change in CO in women with a normal baseline CO profile. However, 31.58% of these women transitioned to either a high or low CO profile (Table S7).

#### 3.3.2. Baseline TPR Profiles

Women with a high baseline TPR profile displayed a significant reduction in TPR following the exercise advice (1760 vs. 1569, *p* = 0.001). No significant change in TPR was observed in women with a low or normal baseline TPR profile (Figure 3 and Table S4).

**Figure 5:**
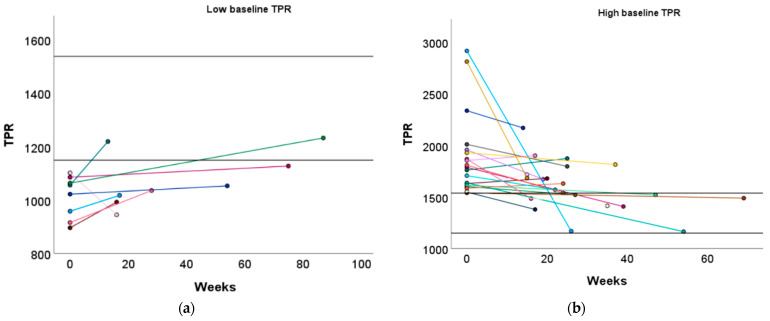
Change in TPR following the preconception physical exercise advice at the subject level. The individual change in TPR between the preconception visit and the post-sport measurement is presented in function of the weeks between the measurements for women with (**a**) a low baseline TPR profile and (**b**) a high baseline TPR profile. Each colored dot represents an individual subject. Each colored line refers to the change in TPR for an individual subject. The black horizontal lines represent the upper and lower limit of the normal reference interquartile range (P25–P75). TPR: total peripheral resistance.

Similar to the high baseline CO group, women with a low baseline TPR profile demonstrated a significant increase in L Aut PI (1.37 vs. 1.55, *p* = 0.029) (Table S4). Although TPR did not significantly increase in women with a low baseline TPR profile, 25.00% of them achieved a normal TPR level after the recommended physical exercise (Figure 5a and Table S8).

Women with a high baseline TPR profile demonstrated a significant increase in ACI (131 vs. 162 1/1.000/s, *p* = 0.030) and a tendency towards an increased VI (60 vs. 66 1/100/s2, *p* = 0.096), which was similar to the low baseline CO group. Additionally, DBP significantly decreased in these women (96 vs. 92 mmHg, *p* = 0.047, Table S4). Furthermore, 42.86% of women with a high baseline TPR profile transitioned to a normal TPR profile (Figure 5b and Table S8).

No significant change in TPR was observed after the physical exercise advice in the women with a normal baseline TPR profile, but 27.27% transitioned to a low TPR profile (Table S8).

### 3.4. Physical Exercise Interval and the Change in CO and TPR

In order to establish whether a more extended period between the PC and PCPS measurement was related to a more pronounced shift in CO and TPR, a correlation analysis was performed (Figures S1 and S2). However, no significant correlation was observed between the change in CO or TPR and the time interval between the PC and PCPS measurements.

## 4. Discussion

The aim of this research was to evaluate whether physical exercise before conception was associated with the normalization of CV dysfunctions that are known to predispose to GHD [5] in non-pregnant women who are at risk for developing GHD in a subsequent pregnancy. The key findings of this study are that (1) distinct CV phenotypes exist prior to conception according to CO and TPR values, similar to the ones observed in pregnancy, and (2) the effects of preconception physical exercise on the CV system depend on the type of the CV phenotype.

### 4.1. Preconception CV Phenotypes

Recent studies have emphasized the importance of maternal CV adaptations during pregnancy [1,2,10,14]. As such, it has been demonstrated that a large subset of women suffering from GHD exhibit disturbed CV adaptations during pregnancy [1,2,3]. Building upon these findings, our research group has already identified distinct abnormal CV phenotypes during various stages of pregnancy that have been associated with different pregnancy outcomes of GHD [2,6]. According to Ohm’s law, MAP is defined by CO × TPR [15]. Consequently, blood pressure is considered to be a spectrum ranging from low CO and high TPR (resistance-dominant) to high CO and low TPR (volume-dominant). Hence, hypertension can result from an excessively high CO, an excessively high TPR, or the combination of both [5]. In essence, we previously observed that women who develop GH begin pregnancy with a lower TPR (Type 1 GH) or a higher CO (Type 2 GH) compared to uncomplicated pregnancies. In contrast, women experiencing EPE initiate pregnancy with a higher TPR and a low/normal CO, whereas those experiencing LPE demonstrate a higher CO and a lower TPR relative to uncomplicated pregnancies. The most significant difference between GH and PE is the lack of abnormal venous Doppler flow measurements in GH. This explains why GH is not associated with symptoms of organ dysfunction, as is the case in PE [3,6].

In the current study, preconception physical exercise did not induce significant changes in CO when observed at the level of the total study population. This observation is consistent with previous studies that have specifically examined the effects of physical exercise on resting CO [16,17]. These studies have consistently shown that regular exercise leads to an increased resting SV and a decreased resting HR, resulting in no significant change in resting CO. However, our analysis also revealed a paradoxical pattern with both improvements in TPR, MAP, and DBP as well as unexplained deteriorations in the L RIVI and the resistivity index of the right uterine artery (R Aut RI) following the advice to engage in physical exercise. To better understand these complex relationships, we conducted a more comprehensive investigation by categorizing subjects according to their baseline CO and TPR values, allowing us to identify distinct preconception CV phenotypes similar to those observed during pregnancy. Interestingly, the impact of physical exercise on these phenotypes varied significantly.

### 4.2. Phenotype-Specific CV Effects

If subjects were categorized according to their baseline CO value, a significant increase/decrease in CO was observed following the preconception physical exercise advice for women with a low/high baseline CO profile, respectively. The same was observed when classified based on their baseline TPR, with a significant decrease in TPR in women with a high baseline TPR value, and a tendency for an increase in TPR in women of the low baseline TPR subgroup. These changes indicate that preconception physical exercise has the potential to normalize CO and TPR levels, thereby promoting the CV system prior to pregnancy. 

#### 4.2.1. Normalization of Aberrant CO and TPR Phenotypes

The decrease in CO among women with a high baseline CO phenotype was accompanied by an increase in HVI. This increased venous wall stiffness reduces venous compliance, restricting venous return, leading to a decrease in preload and subsequently reducing SV and CO, according to Starlings’ law [2,18]. Furthermore, these venous hepatic changes also reflect the importance of the capacitance functioning of the liver [2,19]. Together with the splanchnic veins, the liver constitutes the venous capacitance bed, which stores a large fraction of reserve blood ready to be mobilized in case blood volume levels need to rise quickly (e.g., a fight or flight reaction) [19].

The observed changes in CO and TPR in the low baseline CO and high baseline TPR phenotypes can be attributed to several underlying mechanisms. For example, regular exercise enhances cardiac contractility, leading to an increased CO [16,20]. Additionally, shear stress during physical exercise may stimulate nitric oxide synthesis, resulting in a reduced TPR [21]. Furthermore, ACI and VI were increased in these CV phenotypes after the physical exercise period, reflecting changes in both central and peripheral arterial functions. This finding suggests that physical exercise exerts a favorable effect on the CV system by enhancing cardiac blood outflow. This enhanced blood outflow leads to reduced afterload, contributing to the restoration of CO to normal levels [16].

The tendency for an increase in TPR in women with a low baseline TPR profile after the physical exercise can be attributed to the compensatory response of the body. Women characterized by a low baseline TPR also exhibited a high baseline CO. Upon analyzing their post-sport CV system, a tendency towards a decreased CO was observed. In response to a reduced CO, the body initiates compensatory mechanisms, such as increasing TPR, to maintain the MAP to be within the normal range and ensure adequate blood flow to vital organs [20].

The results described above imply that the impact of physical exercise on volume-dominant CV phenotypes (high baseline CO) was mainly associated with changes in the venous system, while arterial alterations were the main feature observed in resistance-dominant CV phenotypes (high baseline TPR) [2,6]. Although both TPR and DBP significantly reduced in women with a high baseline TPR profile after physical exercise, the mean DBP did not reach the required clinical lower level of 90 mmHg [4]. However, previous research showed that CO and TPR assessments rather than blood pressure are relevant with regard to screening for GHD [5]. The observed significant increase in L Aut PI among women with a high baseline CO or a low TPR profile presents an intriguing aspect that requires further investigation. Although the precise explanation remains elusive, our findings suggest plausible local effects and interactions of physical exercise within the uterine vascular bed. However, it is essential to note that during pregnancy, the normal physiological response is a decrease in L Aut PI [6]. Hence, the importance of the observed increase lies in assessing whether the CV adaptations induced by pregnancy can restore the normal values of this pulsatility index. Further research is needed to elucidate the underlying mechanisms and explore the clinical implications of this finding.

#### 4.2.2. Unchanged CO or TPR Phenotypes

Our study showed no significant changes in CO or TPR after the physical exercise period in women with normal baseline CO or normal TPR profiles, respectively. The lack of change in these parameters is favorable, as the normal CO and TPR values were maintained without any undesirable increase or decrease. However, 31.58% experienced a transition to either a high or low CO profile. Similarly, 27.27% exhibited a transition to a low TPR profile among women with a normal baseline TPR profile. These transitions suggest the dynamic nature of the CV system and highlight individual variations in responses to physical exercise.

### 4.3. Research Novelty

Our research introduces several novel aspects that significantly contribute to extending the knowledge on the impact of physical exercise before conception on the CV system of women who are at risk for GHD in a subsequent pregnancy. Firstly, we focused on investigating the effect of physical exercise on the CV system of non-pregnant women, diverging from previous studies that predominantly focused on pregnant women. Secondly, our research distinguishes itself from others as our research group is the only one measuring the extended aspects of the CV system, including the heart, arterial and venous compartment, and body fluid status. This comprehensive evaluation provides a more holistic understanding of the effects of preconception physical exercise on the CV system. Furthermore, our investigation uniquely considered the existence of distinct preconception CV profiles and evaluated the effect of physical exercise on these specific profiles, thereby enhancing the precision and depth of our findings.

This study represents a pioneering contribution by elucidating the underlying physiological mechanisms through which physical exercise prior to conception mitigates the risk of GHD in subsequent pregnancies for women with volume-dominant or resistance-dominant CV phenotypes, offering novel insights into the field. In essence, our findings serve as a foundation for future studies aiming to develop personalized exercise programs that are tailored to the distinctive preconception CV profile of each woman. This represents a significant shift in maternal healthcare, emphasizing proactive interventions before conception rather than reactive measures during pregnancy. Such a proactive and personalized approach has the potential to improve both short-term and long-term pregnancy outcomes and alleviate the burden of GHD on both mother and fetus.

### 4.4. Limitations of the Study

Our study has several limitations that should be considered when interpreting the results. Firstly, the small sample size within each CV profile group and the lack of a control group limit the generalizability of our findings. Secondly, although we conducted a comprehensive analysis, we did not observe a significant correlation between the change in CO or TPR and the time interval between the PC and PCPS measurements. This suggests that the duration of the physical exercise intervention may not have substantially influenced the observed changes in CO and TPR. Another possible explanation is that the physical exercise effect reaches a plateau faster for some individuals than for others. However, a significant limitation of our study is the lack of objective information regarding the type, intensity, and adherence to the prescribed physical exercise regimen, as well as the baseline physical exercise level of the subjects. This limitation hinders our ability to ascertain the direct impact of preconception physical exercise on the observed changes in CO and TPR. Furthermore, we cannot exclude the differential adherence of subgroups of women to the advised exercise. In addition, 80% of the study population was multiparous and 82.50% experienced previous pregnancy complications, which may affect the way their CV system responds to exercise compared to nulliparous women. 

### 4.5. Future Perspectives

In light of our findings, several future perspectives emerge that can further improve our understanding of the relationship between physical exercise and the preconception CV system. Firstly, it is crucial to conduct larger-scale studies that explore the effect of physical exercise on CV profiles combining CO and TPR in non-pregnant women at risk for GHD. Secondly, the extent to which physiological adaptations induced by pregnancy may counteract or enhance the efforts to bring the preconception CV parameters into a favorable starting position remains unknown. Therefore, future studies should also follow up with these women during pregnancy to better understand the interplay between pregnancy-induced adaptations and the effectiveness of preconception interventions for optimizing the CV system before pregnancy. Thirdly, it is essential that future studies document detailed information on the type, intensity, and adherence of subjects to physical exercise interventions and consider other confounders such as medication adherence, stress levels, and baseline physical exercise level. In addition, more research is needed to evaluate the dose–response relationship between exercise and CV function changes, as well as the different effects of high intensity or resistance training. This will help establish evidence-based guidelines in obstetric care. In the long term, such guidelines may provide a recommendation of the type of exercise, duration, intensity, and other factors best suited for each preconception CV profile, aiming to maximize the effectiveness of physical exercise as a preventive measure against GHD.

## 5. Conclusions

In conclusion, distinct CV phenotypes are already present before conception according to CO and TPR levels, as seen in pregnancy. Additionally, preconception physical exercise induces CV effects in a phenotype-specific way, thereby normalizing CV functions prior to pregnancy. Future research with larger samples and prospective data collection is needed to further explore the relationship between preconception physical exercise and its impact on the complete CV system. Overall, this study offers insights into the potential preventive effect of physical exercise prior to conception and the importance of lifestyle interventions in improving pregnancy and infant outcomes.

## Figures and Tables

**Table 1:** Demographic factors and characteristics of the study population.

	Study Population (n = 40)
**Age (years)**	31.3 (±3.7)
**BMI (kg/m²)**	22.6 (19.8–29.7)
**Parity**	
Nulliparous	8 (20.00%)
Multiparous	32 (80.00%)
**Indication CV assessment**	
Previous pregnancy complications	33 (82.50%)
*GH*	2 (6.06%)
*EPE*	12 (37.50%)
*LPE*	5 (15.63%)
*HELLP*	12 (37.50%)
*IUGR*	12 (37.50%)
*Uncomplicated preterm partus; repeated miscarriages*	2 (6.06%)
Low own birth weight	6 (12.50%)
Familial GHD	2 (2.50%)
**Comorbidity**	18 (45.00%)
Chronic hypertension	12 (30.00%)
Other cardiovascular diseases (cutane vasculitis, arrhythmia)	2 (5.00%)
Thyroid problems	2 (5.00%)
Thrombophilia	2 (5.00%)
Hypercholesterolemia	2 (5.00%)
Kidney problems	1 (2.50%
Diabetes Mellitus	0 (0.00%)
Other (epilepsy)	1 (2.50%)
**Family history of CVD**	16 (40.00%)
**Medication use**	16 (40.00%)
Anti-hypertensive agents	12 (30.00%)
Anti-coagulantia	1 (2.50%)
L-thyroxin	2 (5.00%)
Statins	2 (5.00%)
Anti-epileptica	1 (2.50%)
Other (anti-reflux, folic acid)	1 (2.50%)

Continuous data are presented as a median (IQR) or mean (±SD). Categorical variables are displayed as a number (%). BMI: body-mass index; CV: cardiovascular; GH: gestational hypertension; EPE: early-onset pre-eclampsia; LPE: late-onset pre-eclampsia; HELLP: hemolysis, elevated liver enzymes, low platelet count; IUGR: intrauterine growth restriction; CVD: cardiovascular disease.

## Data Availability

The data presented in this study are available on request from the corresponding author due to ethical and privacy reasons.

## Supplementary Materials

The following supporting information can be downloaded at: https://www.mdpi.com/article/10.3390/jcm13144164/s1, Figure S1: Spearman’s rho correlation between the change in CO and the weeks between the pre-sport and post-sport measurement for women with (a) a high baseline CO profile and (b) a low baseline CO profile; Figure S2: Spearman’s rho correlation between the change in TPR and the weeks between the pre-sport and post-sport measurement for women with (a) a low baseline TPR profile and (b) a high baseline TPR profile; Table S1: Summary of the collected cardiovascular parameters and their biological nature and interpretation; Table S2: The effect of physical exercise on the CV profile of the total study population; Table S3: The effect of physical exercise on the CV profile of women subdivided based on baseline CO value; Table S4: The effect of physical exercise on the CV profile of women subdivided based on baseline TPR value; Table S5: Demographic characteristics of women categorized based on baseline CO level; Table S6: Demographic characteristics of women categorized based on baseline TPR level; Table S7: Cross-tabulation of the group division based on CO at baseline and after the advice to perform physical exercise; Table S8: Cross-tabulation of the group division based on TPR at baseline and after the advice to perform physical exercise.
