# Supplementary material for: Preconception Physical Exercise Is Associated with Phenotype-Specific Cardiovascular Alterations in Women at Risk for Gestational Hypertensive Disorders
Source: J Clin Med. 2024 Jul 16;13(14):4164. doi: 10.3390/jcm13144164 (PMC11277752; doi:10.3390/jcm13144164)
Supplement: Supplementary file 1 [file jcm-13-04164-s001.zip › jcm-3044631-supplementary.pdf]

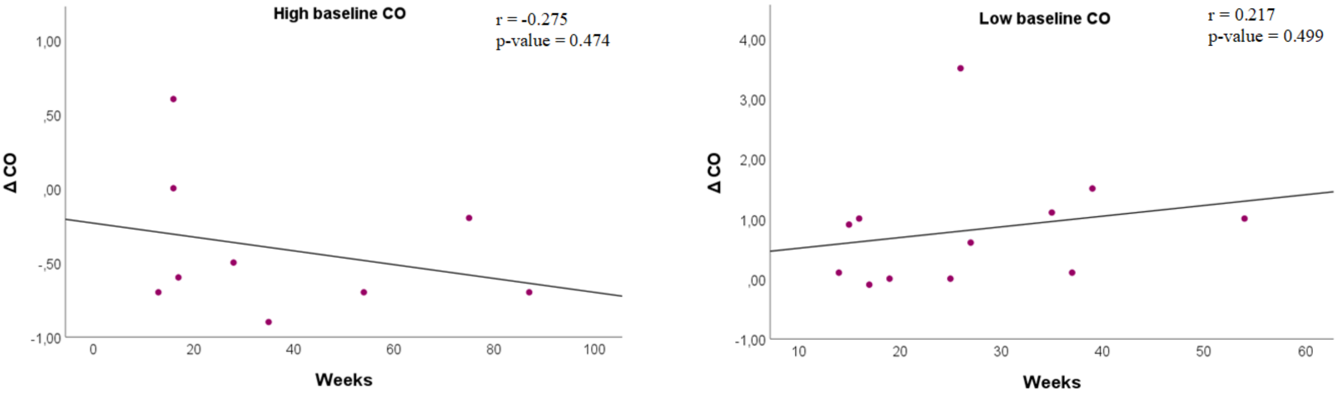

(**a**) (**b**)

Figure S1. Spearman's rho correlation between the change in CO and the weeks between the pre-sport and post-sport measurement for women with (**a**) a high baseline CO profile and; (**b**) a low baseline CO profile. CO: cardiac output. A p-value <0.05 is considered statistically significant.

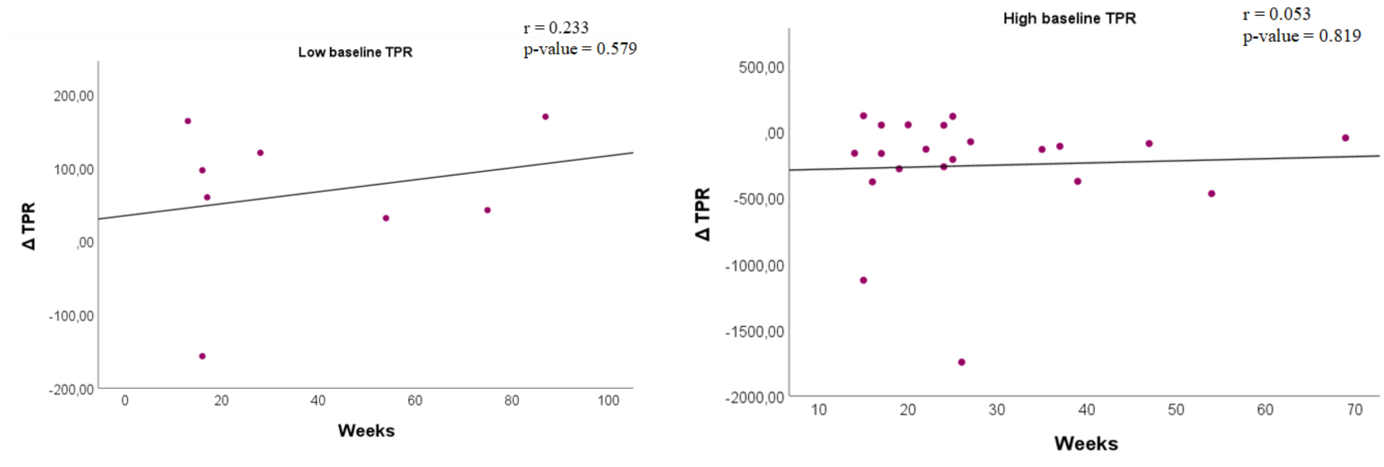

(**a**) (**b**)

Figure S2. Spearman's rho correlation between the change in TPR and the weeks between the pre-sport and post-sport measurement for women with (**a**) a low baseline TPR profile and; (**b**) a high baseline TPR profile. TPR: total peripheral resistance. A p-value <0.05 is considered statistically significant.

Table S1: Summary of the collected cardiovascular parameters and their biological nature and interpretation.

Parameter	Technology	PC reference range	Interpretation
Impedance index (HVI and RIVI)	Doppler ultrasound	HVI: 1.11-1.57<br>L RIVI: 0.33-0.42<br>R RIVI: 0.32-0.44	Reflect the intracycle variability of blood flow velocities in the veins and is directly related to venous tone, i.e., venous wall stiffness. Increased valueas are associated with PE.
Pulse transit time (HVPT and RVPT)	Doppler ultrasound	HVPT: 0.14-0.23 ms<br>L RVPT: 0.23-0.36 ms<br>R RVPT: 0.22-0.36 ms	Inversely related to venous tone, i.e., venous wall stiffness. Decreased values are associated with PE.
Pulsatility index (PI)<br>Resistivity index (RI)	Doppler ultrasound	L Aut PI: 0.92-1.35<br>R Aut PI: 1.03-1.29<br>L Aut RI: 0.63-0.82<br>R Aut RI: 0.68-0.78	Reflects the vascular resistance of the blood flow caused by the microvascular bed distal to the measurement point. Increased values are associated with GHD (EPE).
Body water volumes (TBW, ECW, ICW, ECW/ICW)	Bio-impedance spectrum analysis	TBW: 31.11-36.56 L<br>ECW: 13.15-15.92 L<br>ICW: 17.66-20.46 L<br>ECW/ICW: 0.73-0.79	Provides estimation of the water volumes in the body. Increased values are associated with GHD.
Blood pressure (DBP and MAP)	Impedance cardiography	DBP: 77-93 mmHg<br>MAP: 87-105 mmHg	GHD is characterized by a systolic blood pressure ≥140 mmHg and/or a diastolic blood pressure ≥90 mmHg during pregnancy.
Cardiac output (CO)	Impedance cardiography	CO: 5.0-6.5 L/min	Defines the volume of blood (L) pumped by the heart each minute. Represents one of the key predictors of GHD during pregnancy. Decreased values are associated with EPE and increased values are associated with GH and LPE.
Stroke volume (SV)	Impedance cardiography	SV: 50-100 mL	Defines the volume of blood (mL) pumped out of the left ventricle per beat. SV is directly associated with CO [SVxHR].
Central arterial function (VI and ACI)	Impedance cardiography	VI: 55-81 1/1000/s<br>ACI: 124-191 $1/100/s^2$	Reflects arterial function. Reduced VI and/or ACI reflect abnormal central arterial function.
Total peripheral resistance (TPR)	Impedance cardiography	TPR : 1149-1537 $dyn{\cdot}s{\cdot}cm^{-5}$	TPR represents the overall arterial resistance in the systemic circulation. Represents one of the key predictors of GHD during pregnancy. Calculated using the formula: [MAP × 80/CO]. Increased values are associated with EPE and decreased values are associated with GH and LPE.

HVI: hepatic vein impedance index; RIVI: renal interlobar vein impedance index; L RIVI: left renal interlobar vein impedance index; R RIVI: right renal interlobar vein impedance index; HVPT: hepatic vein pulse transit; RVPT: renal interlobar vein pulse transit; L RVPT: left renal interlobar vein pulse transit; R RVPT: right renal interlobar vein pulse transit; PI: pulsatility index; RI: resistivity index; R aut PI: right uterine artery Doppler pulsatility index; R aut RI: right uterine artery Doppler resistivity index; L aut PI: left uterine artery Doppler pulsatility index; L aut RI: left uterine artery Doppler resistivity index; TBW: total body water volume; ECW: extracellular water volume; ICW: intracellular water volume; ECW/ICW: ratio of ECW to ICW; DBP: diastolic blood pressure; MAP: mean

arterial pressure; CO: cardiac output; SV: stroke volume; VI: velocity index; ACI: aorta flow acceleration index; TPR: total peripheral resistance [3].

Table S2: The effect of physical activity on the CV profile of the total study population.

	PC (N = 40)	PCPS (N = 40)	P-value
**HVPT (ms)**	0.16 (0.13-0.20)	0.15 (0.11-0.21)	0.682
**HVI**	1.35 (0.86-1.58)	1.37 (1.23-1.57)	0.224
**R RVPT (ms)**	0.22 (0.17-0.32)	0.26 (0.16-0.33)	0.568
**R RIVI**	0.51 ± 0.13	0.51 ± 0.11	0.821
**L RVPT (ms)**	0.30 ± 0.09	0.29 ± 0.09	0.329
**L RIVI**	0.45 ± 0.10	0.49 ± 0.13	0.055
**R Aut PI**	1.34 (1.22-1.56)	1.43 (1.28-1.59)	0.194
**R Aut RI**	0.80 (0.71-0.88)	0.84 (0.78-0.89)	0.055
**L Aut PI**	1.37 ± 0.22	1.42 ±0.19	0.202
**L Aut RI**	0.81 (0.76-0.87)	0.85 (0.78-0.89)	0.364
**TBW (L)**	32.73 (30.20-37.43)	31.99 (29.37-37.47)	0.619
**ECW (L)**	14.04 (12.65-16.85)	13.77 (12.17-16.24)	0.962
**ICW (L)**	18.47 (17.23-20.73)	18.14 (16.90-20.61)	0.330
**ECW/ICW**	0.74 (0.72-0.78)	0.75 (0.72-0.78)	0.925
**DBP (mmHg)**	92 ±12	88 ± 9	**0.009**
**MAP(mmHg)**	103 ± 1	99 ± 9	**0.030**
**VI (1/1.000/s)**	61 ± 19	63 ± 15	0.156
**ACI (1/100/s^2)**	139 (104-166)	149 (113-175)	0.108
**LVET (ms)**	239 (221-252)	240 (226-265)	0.224
**CO (L/min)**	5.6 ± 1.3	5.9 ± 1.2	0.064
**TPR (dyn·s·cm^{-5})**	1543 (1247-1780)	1410 (1165-1614)	**0.016**

Data is represented as median (IQR) or mean (± SD). HVPT: hepatic vein pulse transit; HVI: hepatic vein impedance index; R RVPT: right renal interlobar vein pulse transit; R RIVI: right renal interlobar vein impedance index; L RVPT: left renal interlobar vein pulse transit; L RIVI: left renal interlobar vein impedance index; R aut PI: right uterine artery Doppler pulsatility index; R aut RI: right uterine artery Doppler resistivity index; L aut PI: left uterine artery Doppler pulsatility index; L aut RI: left uterine artery Doppler resistivity index; TBW: total body water volume; ECW: extracellular water volume; ICW: intracellular water volume; ECW/ICW: ratio of ECW to ICW; DBP: diastolic blood pressure; MAP: mean arterial pressure; VI: velocity index; ACI: aorta flow acceleration index; LVET: left ventricular ejection time; CO: cardiac output; TPR: total peripheral resistance. A p-value < 0.05 is considered statistically significant.

Table S3: The effect of physical activity on the CV profile of women subdivided based on baseline CO value.

	Low CO			Normal CO			High CO		
	PC (N = 12)	PCPS (N = 12)	P-value	PC (N = 19)	PCPS (N = 19)	P-value	PC (N = 9)	PCPS (N = 9)	P-value
**HVPT (ms)**	0.14 (0.11-0.20)	0.14 (0.10-0.20)	0.875	0.15 (0.13-0.17)	0.15 (0.13-0.21)	0.286	0.22 (0.17-0.31)	0.16 (0.11-0.31)	0.110
**HVI**	1.37 (0.92-1.74)	1.33 (1.22-1.55)	0.937	1.42 (0.86-1.59)	1.41 (1.29-1.60)	0.398	0.91 (0.28-1.40)	1.30 (0.85-1.57)	**0.038**
**R RVPT (ms)**	0.21 (±0.08)	0.25 (±0.10)	0.250	0.25 (±0.09)	0.25 (±0.08)	0.782	0.31 (±0.13)	0.30 (±0.13)	0.840
**R RIVI**	0.52 (±0.11)	0.49 (±0.14)	0.270	0.50 (±0.10)	0.53 (±0.08)	0.254	0.49 (±0.18)	0.50 (±0.12)	0.802
**L RVPT (ms)**	0.25 (±0.07)	0.26 (±0.09)	0.598	0.31 (±0.09)	0.30 (±0.08)	0.470	0.36 (±0.10)	0.31 (±0.11)	0.167
**L RIVI**	0.48 (±0.11)	0.46 (±0.15)	0.601	0.43 (±0.08)	0.50 (±0.12)	**0.017**	0.44 (±0.10)	0.49 (±0.11)	0.151
**R Aut PI**	1.37 (±0.21)	1.31 (±0.26)	0.536	1.28 (±0.23)	1.39 (±0.23)	0.102	1.48 (±0.29)	1.59 (±0.08)	0.286
**R Aut RI**	0.80 (0.72-0.87)	0.79 (0.73-0.87)	0.844	0.79 (0.70-0.82)	0.83 (0.78-0.86)	0.093	0.88 (0.69-0.93)	0.89 (0.87-0.90)	0.213
**L Aut PI**	1.46 (±0.21)	1.36 (±0.21)	0.216	1.31 (±0.23)	1.40 (±0.18)	0.133	1.39 (±0.18)	1.55 (±0.11)	**0.038**
**L Aut RI**	0.87 (0.78-0.90)	0.83 (0.74-0.87)	0.476	0.79 (0.75-0.85)	0.81 (0.78-0.86)	0.136	0.85 (0.78-0.87)	0.87 (0.86-0.90)	0.213
**TBW (L)**	31.02 (29.77-31.39)	30.54 (28.70-31.15)	0.480	32.90 (29.08-38.06)	32.04 (28.44-38.70)	0.968	35.45 (34.48-43.32)	36.62 (33.10-38.80)	0.515
**ECW (L)**	13.12 (12.54-13.71)	12.87 (11.94-13.49)	0.666	14.12 (12.20-17.21)	14.16 (11.64-17.27)	0.494	16.03 (14.85-22.01)	15.87 (14.03-16.82)	0.594
**ICW (L)**	17.68 (17.11-18.11)	17.43 (16.67-18.06)	0.432	18.78 (16.98-21.59)	17.52 (16.48-21.47)	0.365	20.22 (19.33-23.36)	20.75 (19.07-22.12)	0.859

**ECW/ICW**	0.74 (0.71-0.77)	0.73 (0.72-0.77)	0.722	0.73 (0.72-0.78)	0.76 (0.71-0.82)	0.679	0.75 (0.73-0.86)	0.75 (0.73-0.76)	0.594
**DBP (mmHg)**	91 (84-99)	88 (84-90)	0.077	91 (82-99)	90 (82-100)	0.158	86 (84-95)	86 (82-92)	0.286
**MAP(mmHg)**	104 (95-110)	101 (94-102)	0.130	102 (93-110)	100 (91-110)	0.132	99 (92-106)	99 (95-105)	0.953
**VI (1/1.000/s)**	58 (±16)	69 (±14)	**0.042**	63 (±22)	63 (±16)	0.964	58 (±16)	57 (±10)	0.768
**ACI (1/100/s^2)**	121 (112-160)	163 (142-185)	**0.015**	144 (102-188)	149 (98-175)	0.872	132 (82-152)	116 (98-155)	0.953
**LVET (ms)**	237 (221-252)	236 (214-265)	0.784	239 (231-250)	244 (228-267)	0.387	247 (201-269)	228 (219-264)	0.343
**CO (L/min)**	4.4 (4.0-4.5)	4.9 (4.5-5.7)	**0.009**	5.4 (5.2-5.6)	5.5 (5.0-6.5)	0.499	7.5 (7.3-8.0)	7.1 (6.5-8.0)	**0.041**
**TPR (dyn·s·cm^{-5})**	1897 (1673-2254)	1596 (1409-1809)	**0.003**	1529 (1353-1613)	1488 (1207-1569)	0.243	1056 (937-1093)	1053 (1005-1225)	0.086

Continuous data is represented as median (IQR) or mean (± SD). PC: pre-conceptional; PCPS: pre-conceptional post-sport; HVPT: hepatic vein pulse transit; HVI: hepatic vein impedance index; R RVPT: right renal interlobar vein pulse transit; R RIVI: right renal interlobar vein impedance index; L RVPT: left renal interlobar vein pulse transit; L RIVI: left renal interlobar vein impedance index; R aut PI: right uterine artery Doppler pulsatility index; R aut RI: right uterine artery Doppler resistivity index; L aut PI: left uterine artery Doppler pulsatility index; L aut RI: left uterine artery Doppler resistivity index; TBW: total body water volume; ECW: extracellular water volume; ICW: intracellular water volume; ECW/ICW: ratio of ECW to ICW; DBP: diastolic blood pressure; MAP: mean arterial pressure; VI: velocity index; ACI: aorta flow acceleration index; LVET: left ventricular ejection time; CO: cardiac output; TPR: total peripheral resistance. A p-value < 0.05 is considered statistically significant.

Table S4: The effect of physical activity on the CV profile of women subdivided based on baseline TPR value.

	Low TPR			Normal TPR			High TPR		
	**PC (N = 8)**	**PCPS (N = 8)**	**P-value**	**PC (N = 11)**	**PCPS (N = 11)**	**P-value**	**PC (N = 21)**	**PCPS (N = 21)**	**P-value**
**HVPT (ms)**	0.21 (0.17-0.32)	0.19 (0.10-0.32)	0.161	0.15 (0.13-0.16)	0.16 (0.13-0.23)	0.424	0.16 (0.12-0.20)	0.15 (0.11-0.20)	0.986
**HVI**	0.92 (±0.59)	1.14 (±0.46)	0.128	1.27 (±0.36)	1.39 (±0.29)	0.404	1.31 (±0.49)	1.37 (±0.39)	0.589
**R RVPT (ms)**	0.30 (±0.13)	0.30 (±0.18)	0.972	0.23 (±0.09)	0.26 (±0.07)	0.327	0.24 (±0.09)	0.25 (±0.09)	0.729
**R RIVI**	0.50 (0.29-0.69)	0.54 (0.42-0.59)	0.889	0.52 (0.48-0.60)	0.51 (0.47-0.53)	0.139	0.52 (0.39-0.56)	0.55 (0.40-0.60)	0.651
**L RVPT (ms)**	0.36 (±0.11)	0.32 (±0.12)	0.278	0.32 (±0.09)	0.29 (±0.08)	0.205	0.27 (±0.08)	0.28 (±0.09)	0.661
**L RIVI**	0.45 (±0.10)	0.50 (±0.12)	0.246	0.43 (±0.09)	0.49 (±0.09)	0.091	0.45 (±0.10)	0.48 (±0.15)	0.404
**R Aut PI**	1.48 (±0.31)	1.59 (±0.08)	0.340	1.37 (±0.22)	1.38 (±0.28)	0.936	1.29 (±0.22)	1.36 (±0.23)	0.347
**R Aut RI**	0.89 (0.78-0.93)	0.89 (0.86-0.90)	0.400	0.80 (0.70-0.85)	0.83 (0.72-0.88)	0.683	0.79 (0.70-0.84)	0.81 (0.75-0.87)	0.082
**L Aut PI**	1.37 (±0.18)	1.55 (±0.12)	**0.029**	1.41 (±0.22)	1.42 (±0.17)	0.940	1.35 (±0.24)	1.38 (±0.20)	0.644
**L Aut RI**	0.83 (0.77-0.87)	0.88 (0.85-0.90)	0.208	0.83 (0.76-0.88)	0.83 (0.78-0.86)	0.790	0.80 (0.75-0.88)	0.82 (0.74-0.88)	0.322
**TBW (L)**	36.33 (34.46-46.18)	35.62 (33.02-39.42)	0.263	34.03 (29.08-40.00)	32.87 (28.44-38.40)	0.722	31.14 (29.91-35.42)	30.68 (28.72-32.65)	0.768
**ECW (L)**	16.05 (14.80-22.95)	15.15 (13.90-17.00)	0.401	14.36 (12.20-17.37)	14.38 (11.64-16.93)	0.790	13.20 (12.57-14.92)	13.05 (11.97-14.20)	0.651
**ICW (L)**	20.82 (19.15-24.25)	20.48 (19.03-22.42)	0.575	19.67 (16.98-22.63)	17.52 (16.48-20.92)	0.110	17.75 (17.22-19.70)	17.49 (16.75-18.64)	0.821

**ECW/ICW**	0.75 (0.72-0.90)	0.75 (0.72-0.76)	0.674	0.73 (0.71-0.78)	0.76 (0.71-0.90)	0.965	0.74 (0.72-0.78)	0.74 (0.72-0.78)	0.952
**DBP (mmHg)**	87 (±6)	85 (±6)	0.425	88 (±13)	84 (±8)	0.169	96 (±11)	92 (±9)	**0.047**
**MAP(mmHg)**	97 (±8)	98 (±6)	0.616	98 (±13)	94 (±8)	0.109	107 (±12)	102 (±10)	0.066
**VI (1/1.000/s)**	60 (±15)	57 (±11)	0.356	61 (±24)	63 (±18)	0.575	60 (±18)	66 (±14)	0.096
**ACI (1/100/s^2**	136 (92-157)	117 (109-159)	1.000	143 (82-188)	149 (87-173)	0.859	131 (107-166)	162 (136-190)	**0.030**
**LVET (ms)**	248 (199-275)	234 (226-270)	0.401	237 (231-250)	249 (235-273)	**0.045**	238 (222-251)	232 (216-256)	0.728
**CO (L/min)**	7.6 (7.2-8.2)	7.3 (6.6-8.1)	0.073	5.5 (5.3-5.9)	5.8 (5.1-6.8)	0.646	4.5 (4.4-5.3)	5.1 (4.6-5.9)	**0.025**
**TPR (dyn·s·cm^{-5})**	1039 (926-1079)	1044 (998-1196)	0.093	1374 (1329-1511)	1366 (1117-1456)	0.534	1760 (1600-1941)	1569 (1448-1756)	**0.001**

Continuous data is represented as median (IQR) or mean (± SD). PC: pre-conceptional; PCPS: pre-conceptional post-sport; HVPT: hepatic vein pulse transit; HVI: hepatic vein impedance index; R RVPT: right renal interlobar vein pulse transit; R RIVI: right renal interlobar vein impedance index; L RVPT: left renal interlobar vein pulse transit; L RIVI: left renal interlobar vein impedance index; R aut PI: right uterine artery Doppler pulsatility index; R aut RI: right uterine artery Doppler resistivity index; L aut PI: left uterine artery Doppler pulsatility index; L aut RI: left uterine artery Doppler resistivity index; TBW: total body water volume; ECW: extracellular water volume; ICW: intracellular water volume; ECW/ICW: ratio of ECW to ICW; DBP: diastolic blood pressure; MAP: mean arterial pressure; VI: velocity index; ACI: aorta flow acceleration index; LVET: left ventricular ejection time; CO: cardiac output; TPR: total peripheral resistance. A p-value < 0.05 is considered statistically significant.

Table S5: Demographic characteristics of women categorized based on baseline CO level.

	Low CO<br>N = 12	Normal CO<br>N = 19	High CO<br>N = 9	P^{N-L}	P^{N-H}	P^{L-H}
**Age (years)**	31.3 (±3.8)	31.4 (±4.1)	31.1 (±2.7)	0.996	0.984	0.996
**BMI (kg/m²)**	19.8 (18.5-22.3)	22.3 (20.0-26.8)	31.6 (27.6-33.5)	0.249	**0.035**	**<0.001**
**Parity**				0.653	1.000	1.000
Nulliparous	3 (25.00%)	3 (15.79%)	2 (22.22%)			
Multiparous	9 (75.00%)	16 (84.21%)	7 (77.78%)			
**Indication CV measurement**						
Previous pregnancy complications	9 (75.00%)	16 (84.21%)	7 (77.78%)	0.653	1.000	1.000
Low own birth weight	3 (25.00%)	3 (15.79%)	0 (0.00%)	0.653	0.207	0.229
Familial GHD	0 (0.00%)	1 (5.26%)	1 (11.11%)	1.000	1.000	0.429
**Previous pregnancy complication**						
GH	0 (0.00%)	2 (10.53%)	0 (0.00%)	0.510	1.000	/
EPE	3 (25.00%)	7 (36.84%)	2 (22.22%)	0.697	0.670	1.000
LPE	3 (25.00%)	0 (0.00%)	2 (22.22%)	0.049	0.095	1.000
HELLP	4 (33.33%)	6 (31.57%)	2 (22.22%)	1.000	1.000	0.659
IUGR	3 (25.00%)	8 (42.11%)	1 (11.11%)	0.452	0.195	0.603
Uncomplicated preterm partus; repeated miscarriages	0 (0.00%)	0 (0.00%)	1 (11.11%)	/	0.321	0.429
**Comorbidity**						
Chronic hypertension	4 (33.33%)	7 (36.84%)	1 (11.11%)	1.000	0.214	0.338
Other cardiovascular diseases (cutane vasculitis, arrhythmia)	1 (8.33%)	0 (0.00%)	0 (0.00%)	0.387	/	1.000
Thyroid problems	0 (0.00%)	2 (10.53%)	0 (0.00%)	0.510	1.000	/
Thrombophilia	2 (16.67%)	0 (0.00%)	0 (0.00%)	0.142	/	0.486
Hypercholesterolemia	0 (0.00%)	1 (5.26%)	1 (11.11%)	1.000	1.000	0.429
Kidney problems	0 (0.00%)	1 (5.26%)	0 (0.00%)	1.000	1.000	/
Other (epilepsy)	1 (8.33%)	0 (0.00%)	0 (0.00%)	0.387	/	1.000
**Family history of CVD**	4 (33.33%)	9 (47.36%)	3 (33.33%)	0.166	0.774	0.211

**Medication use**						
Anti-hypertensive agents	5 (41.67%)	7 (36.84%)	0 (0.00%)	1.000	0.062	0.045
Anti-coagulantia	0 (0.00%)	0 (0.00%)	1 (11.11%)	/	0.321	0.429
L-thyroxin	0 (0.00%)	2 (10.53%)	0 (0.00%)	0.510	1.000	/
Statins	0 (0.00%)	1 (5.26%)	1 (11.11%)	1.000	1.000	0.429
Anti-epileptica	1 (8.33%)	0 (0.00%)	0 (0.00%)	0.387	/	1.000
Other (anti-reflux, folic acid)	0 (0.00%)	0 (0.00%)	1 (11.11%)	/	0.321	0.429

Continuous data is represented as median (IQR) or mean (± SD). Categorical variables are displayed as n (%). CV: cardiovascular; EH: essential hypertension; GH: gestational hypertension; EPE: early-onset preeclampsia; LPE: late-onset preeclampsia; HELLP: hemolysis, elevated liver enzymes, low platelet count; IUGR: intrauterine growth restriction. The level of significance for differences between groups is indicated by P^{N-L}: normal versus low, P^{N-H}: normal versus high, P^{L-H}: low versus high. For continuous data, a p-value < 0.050 is considered statistically significant. Because the chi-square test does not take into account multiple testing, the significance level for categorical data was made more stringent i.e. a p-value <0.017 is considered statistically significant.

Table S6: Demographic characteristics of women categorized based on baseline TPR level.

	Low TPR N = 8	Normal TPR N = 11	High TPR N = 21	P^{N-L}	P^{N-H}	P^{L-H}
**Age (years)**	31.4 (±2.7)	31.1 (±4.0)	31.3 (±3.9)	0.985	0.983	1.000
**BMI (kg/m²)**	31.0 (26.4-32.6)	22.7 (20.2-35.1)	20.1 (18.7-24.0)	0.412	0.060	**<0.001**
**Parity**				0.546	0.637	0.947
Nulliparous	2 (25.00%)	1 (9.09%)	5 (23.81%)			
Multiparous	6 (75.00%)	10 (90.91%)	16 (76.19%)			
**Indication CV measurement**						
Previous pregnancy complications	6 (75.00%)	10 (90.91%)	16 (76.19%)	0.348	0.311	0.947
Low own birth weight	0 (0.00%)	1 (9.09%)	5 (23.81%)	1.000	0.637	0.283
Familial GHD	1 (12.50%)	0 (0.00%)	1 (4.76%)	0.421	1.000	0.483
Familial EH	0 (0.00%)	1 (9.09%)	0 (0.00%)	0.381	0.160	1.000
**Previous pregnancy complication**						
GH	0 (0.00%)	1 (9.09%)	1 (4.76%)	1.000	1.000	1.000
EPE	2 (25.00%)	4 (36.36%)	6 (28.57%)	1.000	0.703	1.000
LPE	2 (25.00%)	0 (0.00%)	3 (14.28%)	0.164	0.534	0.597
HELLP	1 (12.50%)	4 (36.36%)	7 (33.33%)	0.338	1.000	0.381
IUGR	1 (12.50%)	4 (36.36%)	6 (28.57%)	0.338	1.000	0.381
Uncomplicated preterm partus; repeated miscarriages	1 (12.50%)	0 (0.00%)	0 (0.00%)	0.421	/	0.276
**Comorbidity**	2 (25.00%)	3 (27.27%)	13 (61.90%)			
Chronic hypertension	1 (12.50%)	3 (27.27%)	8 (38.09%)	0.603	0.703	0.371
Other cardiovascular diseases (cutane vasculitis, arrhythmia)	0 (0.00%)	0 (0.00%)	1 (4.76%)	/	1.000	1.000
Thyroid problems	0 (0.00%)	1 (9.09%)	1 (4.76%)	1.000	1.000	1.000
Thrombophilia	0 (0.00%)	0 (0.00%)	2 (9.52%)	/	0.534	1.000
Hypercholesterolemia	1 (12.50%)	0 (0.00%)	1 (4.76%)	0.421	1.000	0.483
Kidney problems	0 (0.00%)	1 (9.09%)	0 (0.0%)	1.000	0.344	/
Other (epilepsy)	0 (0.00%)	0 (0.00%)	1 (4.76%)	/	1.000	1.000

**Family history of CVD**	2 (25.00%)	4 (36.36%)	10 (47.62%)	1.000	0.712	0.408
**Medication use**						
Anti-hypertensive agents	0 (0.00%)	3 (27.27%)	9 (42.86%)	0.228	0.465	0.033
Anti-coagulantia	1 (12.50%)	0 (0.00%)	0 (0.00%)	0.421	/	0.276
L-thyroxin	0 (0.00%)	1 (9.09%)	1 (4.76%)	1.000	1.000	1.000
Statins	1 (12.50%)	0 (0.00%)	1 (4.76%)	0.421	1.000	0.483
Anti-epileptica	0 (0.00%)	0 (0.00%)	1 (4.76%)	/	1.000	1.000
Other (anti-reflux, folic acid)	1 (12.50%)	0 (0.00%)	0 (0.00%)	0.421	/	0.276

Continuous data is represented as median (IQR) or mean (± SD). Categorical variables are displayed as n (%). CV: cardiovascular; EH: essential hypertension; GH: gestational hypertension; EPE: early-onset preeclampsia; LPE: late-onset preeclampsia; HELLP: hemolysis, elevated liver enzymes, low platelet count; IUGR: intrauterine growth restriction. The level of significance for differences between groups is indicated by P^{N-L}: normal versus low, P^{N-H}: normal versus high, P^{L-H}: low versus high. For continuous data, a p-value < 0.050 is considered statistically significant. Because the chi-square test does not take into account multiple testing, the significance level for categorical data was made more stringent i.e. a p-value <0.017 is considered statistically significant.

Table S7: Cross-tabulation of the group division based on CO at baseline and after the advice to perform physical activity.

		PCPS			
		**Low CO**	**Normal CO**	**High CO**	**Total**
**PC**	**Low CO**	6	6	0	12
	**Normal CO**	3	13	3	19
	**High CO**	0	3	6	9
	**Total**	9	22	9	40

CO: cardiac output; PC: pre-conceptional; PCPS: pre-conceptional post-sport.

Table S8: Cross-tabulation of the group division based on TPR at baseline and after the advice to perform physical activity.

		PCPS			
		**Low TPR**	**Normal TPR**	**High TPR**	**Total**
**PC**	**Low TPR**	6	2	0	8
	**Normal TPR**	3	8	0	11
	**High TPR**	0	9	12	21
	**Total**	9	19	12	40

TPR: total peripheral resistance; PC: pre-conceptional; PCPS: pre-conceptional post-sport.
